# Invasive Parasites, Habitat Change and Heavy Rainfall Reduce Breeding Success in Darwin's Finches

**DOI:** 10.1371/journal.pone.0107518

**Published:** 2014-09-23

**Authors:** Arno Cimadom, Angel Ulloa, Patrick Meidl, Markus Zöttl, Elisabet Zöttl, Birgit Fessl, Erwin Nemeth, Michael Dvorak, Francesca Cunninghame, Sabine Tebbich

**Affiliations:** 1 Department of Behavioural Biology, University of Vienna, Vienna, Austria; 2 Charles Darwin Foundation, Puerto Ayora, Santa Cruz Island, Galápagos, Ecuador; 3 Institute for Science and Technology, Klosterneuburg, Austria; 4 Department of Zoology, University of Cambridge, Cambridge, United Kingdom; 5 BirdLife Austria, Vienna, Austria; Hungarian Academy of Sciences, Hungary

## Abstract

Invasive alien parasites and pathogens are a growing threat to biodiversity worldwide, which can contribute to the extinction of endemic species. On the Galápagos Islands, the invasive parasitic fly *Philornis downsi* poses a major threat to the endemic avifauna. Here, we investigated the influence of this parasite on the breeding success of two Darwin's finch species, the warbler finch (*Certhidea olivacea*) and the sympatric small tree finch (*Camarhynchus parvulus*), on Santa Cruz Island in 2010 and 2012. While the population of the small tree finch appeared to be stable, the warbler finch has experienced a dramatic decline in population size on Santa Cruz Island since 1997. We aimed to identify whether warbler finches are particularly vulnerable during different stages of the breeding cycle. Contrary to our prediction, breeding success was lower in the small tree finch than in the warbler finch. In both species *P. downsi* had a strong negative impact on breeding success and our data suggest that heavy rain events also lowered the fledging success. On the one hand parents might be less efficient in compensating their chicks' energy loss due to parasitism as they might be less efficient in foraging on days of heavy rain. On the other hand, intense rainfalls might lead to increased humidity and more rapid cooling of the nests. In the case of the warbler finch we found that the control of invasive plant species with herbicides had a significant additive negative impact on the breeding success. It is very likely that the availability of insects (i.e. food abundance)is lower in such controlled areas, as herbicide usage led to the removal of the entire understory. Predation seems to be a minor factor in brood loss.

## Introduction

Invasive alien pathogens and parasites are a major and growing threat to biodiversity worldwide. Small host populations of endemic species are particularly vulnerable because extinction can occur before the hosts have a chance to evolve effective defences [1], [2]. This is especially true if the parasite is a generalist because it can switch to another host if it has driven one close to extinction [3]. The adverse effect of introduced parasites has been documented in a range of avian species (reviewed in [2]). A famous example is the extinction of most of the endemic Hawaiian honeycreepers because of the introduction of avian malaria and its vector [4]. The avifauna of almost all islands of the Pacific Ocean has been dramatically altered by introduced species or other human impact [5]. In this respect the Galápagos archipelago is one of the exceptions, as no bird species has become extinct in modern times. This archipelago hosts the endemic Darwin's finches which have provided the inspiration for some of the most important ideas in evolutionary biology. However, the introduction of predators, pathogens, and parasites have led to increasing pressures affecting several Darwin's finch species [6]–[10].

One of the biggest threats to the Galápagos avifauna is the obligate bird parasite *Philornis downsi* [11], which was first discovered on the archipelago in 1997 [12]. Adult flies lay their eggs in bird nests where the parasitic larvae then hatch and suck blood from the nestlings [13]. Correlative as well as experimental studies have shown that *P. downsi* has a negative impact on nestling growth, haemoglobin levels, and fledgling success [14]–[18]. The influence of parasitism on breeding success in Darwin's finches is highly variable from year to year, and mortality ranges from 16 to 95% in all finch nests (reviewed in [18], [19]). The reasons for this high variation are poorly understood so far but could stem from differences in precipitation between years [20], but also see [21]. The Galápagos archipelago is characterised by a highly seasonal climate with pronounced wet and dry seasons, as well as extraordinary yearly variation in rainfall [22]. Years with intense rainfalls (El Niño years) and severe drought years reoccur at irregular intervals. However, within the wet season there is also variation in precipitation, which has received little attention so far. Rainfall can reach 50 ml and more on some days, whereas on other days no precipitation is measured. An interaction between rainfall and *P. downsi* prevalence and intensity has been found in *Philornis* sp. which affect various bird species in Puerto Rico [23] and Argentina [24].

The highest prevalence and intensity (larvae per nest or chick; [25]) of *P. downsi* infestation on the Galápagos Islands was found on Santa Cruz Island [26]. On this island, several insectivorous passerines declined between 1997 and 2010 [27]. The greatest decline was observed in the warbler finch (*Certhidea olivacea*). This insectivorous species is an arboreal finch and is the smallest of the Darwin's finches. It is restricted to the highlands, which were previously covered by the endemic humid *Scalesi*a forest. During the last century this forest has to a large extent been transformed into agricultural areas. The warbler finch population has shrunk by 50% in the *Scalesia* forest and by up to 75% in the agricultural areas [27]. The closely related grey warbler finch (*Certhidea fuscua*) has already gone extinct on Floreana Island as there are no records of this species since more than sixty years [28]. Other than parasitism, the loss of primary habitat could be a possible reason for the population decline of the warbler finch. By 2009, the *Scalesia* forest, which holds the highest density of warbler finches [27], was reduced to only 2% of its original area [29]. Additionally, introduced trees and shrubs have invaded these remnant *Scalesia* forest patches [30], [31]. The *Scalesia* forests are most affected by the introduced plant species *Rubus niveus*. To control this invasive neophyte, the National Park uses strong herbicides which lead to the temporary removal of the entire understory. We hypothesize that this dramatic habitat change may lead to changes in plant communities and species composition of invertebrates, which could also negatively influence the insectivorous bird species. Finally, introduced predators, especially rats (*Rattus rattus*), may negatively affect bird species [32]. Black rats (*Rattus rattus*) and Norway rats (*Rattus norvegicus*) have been introduced on all inhabited Galápagos Islands [33]. Due to their climbing skills and omnivorous diet, the black rats in particular are considered a threat to the Galápagos avifauna [10], [19], [34], [35]. A study on mangrove finches found that rats can cause significant brood loss [10]. Furthermore, rats may have a stronger influence on the warbler finch than the other finch species, as warbler finch nests are built lower down in the canopy [17].

A population decline may be caused by high adult mortality or low recruitment. Therefore, one possible factor in the decline of the warbler finch on Santa Cruz Island is low breeding success. Thus, the aim of the study was to measure the breeding success of the warbler finch and to identify the reasons for brood loss. We compared the breeding success of the warbler finch, whose populations declined, with the breeding success of the sympatric small tree finch, whose population remained stable during the respective time period [27]. We predicted that warbler finches suffer a higher brood loss due to *P. downsi* than small tree finches because they showed a higher parasite intensity than expected for their body size and nest size in previous studies [17]. Furthermore, we predicted that warbler finches suffer a higher brood loss due to predation (by rats) because their nests are built lower in the vegetation than those of small tree finches (warbler finch: 4.2 m, small tree finch: 6.2 m [17]). In addition, we predicted lower breeding success in areas where herbicides were used to control invasive plant species because both species may be negatively affected by reduced insect availability during chick rearing.

## Methods

### Study site

The study was conducted during the main breeding season (January to the end of March 2010 and 2012) in the highland area around Los Gemelos (0°37′34″ S, 90°23′10″ W) on Santa Cruz Island, Galápagos. This area is dominated by the endemic tree *Scalesia pedunculata* and is thus called the *Scalesia* zone. On 11 ha of our 30 ha study site, the National Park applied strong herbicides to control the invasion of *R. niveus*, which results in the removal of the whole forest understory. The procedure of *Rubus* control consisted in manually cutting down all introduced and invasive plant species and then regularly treating the area with herbicides to prevent re-growth of neophytes.

### Population survey

Our data collection is part of a population survey of the two focal species, warbler finch and small tree finch, that started in the *Scalesia* zone in 1997. We conducted point counts with unlimited distance following Dvorak et al. [27]. Data of the population survey in 2010 are already published (see [27]). In 2012, 26 points were counted once from February 18 to 21 by Michael Dvorak and Birgit Fessl. Each point count lasted 5 minutes and was conducted between 06:30 and 10:30 am, which is when Darwin's finches show the highest singing activity. The 26 points were spaced at least 70 m apart. The number of all singing birds (presumed to be territory holding males) and the distance of each singing individual to the observer were recorded for each point. However, in order to measure the population trends over the whole period from 1997 to 2012, only the number of singing males per point was used.

In 2012, we also recorded the age structure of the small tree finch population in the *Scalesia* forest. The brownish head coloration of young small tree finch males turns black in a specific moulting pattern as the subjects age [36]. Although six distinct moulting patterns were identified, interobserver reliability of plumage classification was low for some categories [36].Thus, each small tree finch male found displaying near its nest (irrespective if it was already paired or not) was assigned to one of two categories following Kleindorfer's [36] coloration categories: (i) young males, whose black plumage is maximally extended from around the beak to the eye region, and (ii) old males, whose black plumage has already extended to the throat and the back part of the head and nape.

### Parasite life cycle

While *P. downsi* is an obligate bird parasite in its three larval stages, adult flies feed on organic matter [13], [38]. The first instar larvae usually develop within the chicks' nares, although they were also found freely moving in the nest material ([18], Cimadom personal observation). First instar larvae cause beak malformations in the developing chick, which can persist into adulthood [37]. The second and third instar larvae are found in the bottom layer of the nest and feed on nestling blood and tissue during the night [13], [38], [39]. The larvae pupate at the bottom of the nest and emerge as adult flies approximately 10 to 14 days later ([38], Cimadom personal observation). Bird nests are often subject to multiple infections by *P. downsi* [40].

### Nest monitoring and parasite collection

We monitored the nests of both Darwin's finch species – warbler finch and small tree finch – in an area of approximately 30 ha within the *Scalesia* forest. Nest status was checked at different intervals depending on the current breeding status to determine onset of breeding, number of eggs, hatching day, number of nestlings and date of failure or fledging. Intervals of nest observations varied depending on breeding status to minimize disturbance and optimize accuracy of information. During nest building nests were checked at 5 days interval, during incubation at 3 days interval, during feeding at 2 days interval and close to fledging daily. We inspected the inside of the nests with a video camera in 2010 and a small endoscopic video camera (dnt Findoo 3.6) in 2012, which allowed recordings of clutch as well as brood size (only in 2012). After each nest inspection, we waited until the parents resumed incubation or feeding, which occurred in all instances. Therefore we are confident that filming did not lead to abandonment of nests. Successful nests (breeding success) were defined as nests that produced at least one fledgling. Nests that failed were assigned to the following categories: (1) abandoned (pair stopped incubating, no chicks but eggs still present in the nest), (2) dead chicks (all chicks dead but still in the nest), (3) empty nest (previously active nest, chicks ≤6 days), These nests showed no clear signs of predation but predation cannot be excluded. Alternatively dead chicks may have been thrown out by parents [14]. (4) Predated (nest destroyed, with clear signs of predation or intact and empty, previously contained chicks ≥7 days – assumed to be too big to be thrown out by parents), and (5) others (e.g. nesting tree collapsed, nest fallen down due to wind).

Because of the smaller size of the endoscopic camera, the quality of the pictures was much better and allowed reliable age estimates. Therefore, we included only the data set of 2012 in analysis that included chick age, date of starting incubation and hatching date. The chicks' age (in days) in successful and failed nests was calculated by combining information from nest inspections with a series of images of growing nestlings (when inspections coincided with hatching). The hatching date was calculated as the last observation date minus chick age. Where possible, the calculated hatching date was cross-checked with observational data from nest inspections.

In total, 43 nests were found before incubation started and were successfully incubated until the chicks' hatched. The incubation time was calculated for each nest as the time period (in days) from date of first incubating observation to hatching date. The mean incubation time was 13.7±2.0 SD days (n = 27, median 14) for the warbler finch and 13.6±2.0 SD days (n = 16, median 14) for the small tree finch. Thus, for all nests, the date when incubation began was defined as the hatching date minus 14 days. For nests that had been found before incubation started and were abandoned during incubation, the first observation of incubation was used as starting date of incubation.

All monitored nests were collected in separate sealed plastic bags after nesting failure or after the chicks fledged. Eggs found in failed nests were inspected to identify the developmental stage of the embryo (undeveloped: no signs of developed embryo, developed embryo: first tissues of small embryo visible, and developed embryo close to hatching: embryo showing feathers and nest monitoring suggested end of incubation). Nests were later dismantled in the laboratory in order to count *P. downsi* larvae, pupae and empty puparia. *P. downsi* intensity per nest was defined as the total number of *P. downsi* specimens per nest (after [25]). *P. downsi* intensity per chick was calculated by dividing *P. downsi* intensity per nest by the number of chicks of a given nest. However, as brood size was not known for all nests in 2010, *P. downsi* intensity per nest was used for species and year comparisons of *P. downsi* infestation. We are well aware that brood size plays an important role as the detrimental impact of *P. downsi* parasitism increases with decreasing brood size, but (1) we are interested in the general infestation pattern of the two species over the years and (2) there is no difference in clutch sizes between the two species (warbler finch: 2.43±0.5, mean ± SD, n = 56; small tree finch: 2.46±0.5, n = 48; Mann-Whitney test: Z_102_ = −0.303, p = 0.844) and (3) there is no indication that clutch size differs between the two breeding seasons. As the number of parasites can only be counted by destroying the nest, parasite abundance was assigned to the age of the chicks at the time breeding activity terminated at a given nest. In our data parasite intensities increase with chick age (but see [20]) because of multiple infections of nests with *P. downsi* [40]. This leads to the contradictory finding that successful nests have the highest *P. downsi* intensities. Since we cannot asses *P. downsi* intensity of successful nests prior to fledging and thus cannot compare them to failed nests of the same age, *P. downsi* intensity could not predict breeding success and thus was excluded from further analysis.

### Habitat parameters and daily rainfall

In 2012 we estimated nesting height, maximum and average canopy height, as well as vegetation cover of herb-layer, bush-layer (including *R. niveus*), *R. niveus* separately and canopy-layer (vegetation>3 m), of the surrounding area (5 m radius) for each active nest encountered. Height levels were estimated in one-meter intervals and vegetation covers in 10 percent intervals. Furthermore, the nesting location was assigned to (i) *Rubus* controlled sites (area of 5 m radius around the nest,still showed clear signs of herbicide usage) or (ii) not controlled sites (no signs of herbicide usage detected, usually dense understory).

Data of daily rainfall were provided by the Charles Darwin Station from the meteorological station near Santa Rosa (0°39′16,45″ S, 90°24′12,96″ W, elevation 500 m a.s.l., about 3.5 km from our study site) for 2012. Within the relevant study period of 101 days in 2012 (date of starting incubation at the first nest to the date of the last nest inspection), it rained more than 10 mm per day on 26 days. These days were classified as “heavy rain days”.

### Estimation of rat predation

In March 2012, we conducted an artificial nest experiment to identify potential nest predators and estimate nest predation rates following the method developed by Fessl et al. [10]. The artificial nests were made from coconut fibre. Each nest contained two Plasticine eggs (Acrilex modelling clay) resembling finch eggs dipped in egg albumen, which were tied to the nest. All nests and eggs were handled with surgical gloves to avoid them being tainted with human odour. A total of 30 artificial nests were systematically placed along two transects (15 nests per transect), with a minimum distance of at least 30 m between nests, and about 3 m height, mainly in *Scalesia* trees. To examine the effect of *Rubus* control (understory removal), one transect was located in a controlled area with a 5 m bush cover around the artificial nest of 21%±27 (mean ± SD), whilst the other was in a non-controlled area with a bush cover of 87%±15 (mean ± SD). Nests were revisited after 3, 6 and 9 days before being removed. Eggs with possible signs of predation were removed from nests and replaced with new Plasticine eggs, resulting in a total of 180 possible egg predation events. Traces on artificial eggs were compared to marks left by a rat's dental impression or by a bird's beak and assigned to (1) rats, if clear rat tooth marks were present, (2) birds, if single punctures were visible, or (3) unidentified. We calculated the percentage of predated individual nests over the whole 9-day period for each transect. Furthermore, we measured the number of predated eggs and, of these eggs, calculated the percentage that had been predated by rats.

### Analysis

As breeding pairs did re-nest within one breeding season, especially after a breeding failure, it is likely that we monitored different nests from the same breeding pair. Nests found in close proximity of a monitored nest, where a breeding attempt had recently terminated, were considered to belong to the same breeding pair. To reduce such pseudo-replication, only one randomly chosen nest per breeding pair was included in the analysis. We monitored a total of 204 active nests and excluded 13 nests because of possible re-nesting, resulting in a dataset of 191 nests. In 2010 we monitored 30 warbler finch nests and 32 small tree finch nests, and in 2012, 74 warbler finch nests and 55 small tree finch nests were observed. To assess variation between years, we conducted a separate analysis for breeding success and *P. downsi* infestation for 2010 and 2012.

To assess population trends for both species, non-parametric correlations and t-tests were calculated to compare bird numbers over specific years. To compare different distributions (e.g. age structure of the small tree finch population at different time periods, species differences in breeding success, breeding success and predation of artificial nests in *Rubus* controlled compared with uncontrolled sites), we either computed Chi^2^-tests or Fisher exact tests (when the sample size was too small for Chi^2^-tests).

To compare *P. downsi* abundance between species and years, we calculated a two-way ANOVA. Species differences in *P. downsi* intensity between age classes were tested with two-tailed Student t-tests. To make the data set comparable to previous studies, we distinguished two age classes according to Dudaniec et al. [20]. For the multivariate analyses of the breeding success in 2012, we constructed Generalized Linear Models (GLMs) with binomial error structure and a logit-link function. Successful nests were defined as nests with at least one fledged young, while all other nesting attempts were counted as failures. Since stepwise methods may produce inflated Type I errors, we present full models with all variables entered [41] and reduced models that retained only the significant predictor variables. To avoid multi-colinearity [42], we chose among predictor variables that were highly correlated (Spearman rank correlation r>0.4), the one that seemed to be of higher biological relevance. Predicted variables were breeding success (fledged young or not), predictor variables were height of the nest (m), cover of canopy (%), the day-of-year of the start of incubation (ordinal date), and the percentage of heavy rain days during the incubation and nestling period. We tested the effect of *Rubus* control on the breeding success separately using Chi^2^-tests because of inter-correlation with other habitat variables and low sample size. Statistical analyses were done with R 3.0.1 (R Development Core Team 2013).

### Ethics statement

The study was conducted in the protected areas of the Galápagos National park. Permission to conduct this study was granted by the Galápagos National Park and the Charles Darwin research station (Project: PC-54-11, Permit Nr. PR.CDS.ACI.P01.R02). As our study was purely descriptive, strictly non-invasive, and based exclusively on behavioural observations, they are classified as non-animal experiments in accordance with the Austrian Animal Experiments Act (§ 2. Federal Law Gazette No. 501/1989).

## Results

### Population survey

**Figure 1:**
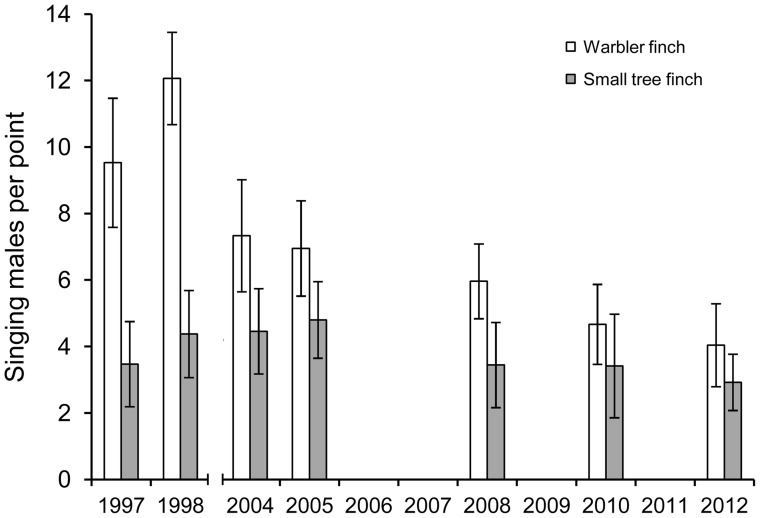
Population trends of warbler finches and small tree finches. Mean (± SD) number of singing warbler finch males and small tree finch males per point count in the *Scalesia* zone on Santa Cruz, Galápagos, for the years 1997, 1998, 2008, 2010 (data from [27]), 2004 and 2005 (Dvorak et al. unpublished data) and 2012.

**Figure 2:**
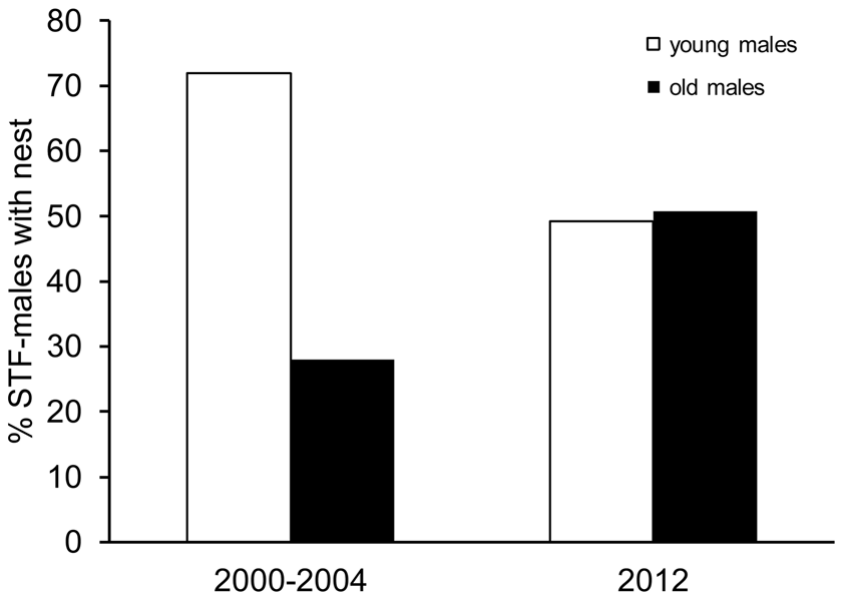
Age structure of the small tree finch population. Percentage of young and old displaying small tree finch (STF) males of the *Scalesia* zone population in 2000–2004 (n = 132, [36]) and 2012 (n = 63) on Santa Cruz, Galápagos.

Point counts showed that the warbler finch population decreased significantly over the last 15 years (Spearman's Rho_5_ = −0.964, n = 7, p<0.01, data from [27] and Dvorak et al. unpublished data). Compared to 1997, the number of singing males dropped significantly by 58% (two tailed Student t-test: n_1997_ = 17, n_2012_ = 26, t_41_ = 11.318, p<0.001; Figure 1). In contrast to the warbler finch, the small tree finch population did not decrease significantly over the whole period from 1997 to 2012 (Spearman's Rho_5_ = −0.643, n = 7, p = 0.12). Instead, there was an initial significant increase from 1997 to 2005 by 38% (two-tailed Student t-test: n_1997_ = 17, n_2005_ = 20, t_35_ = 3.324, p = 0.002, data from [27] and Dvorak et al. unpublished data) followed by a significant decrease by 39% until 2012 (two-tailed Student t-test: n_2005_ = 20, n_2012_ = 26, t_44_ = 6.380, p<0.001, Figure 1). Additionally, the age structure of the small tree finch population significantly changed between 2000–2004 [36] and 2012 (Chi^2^-test: χ^2^_1, n = 195_ = 10.396, p = 0.001; Figure 2). The population from 2000–2004 consisted of 72% “young” males and 28% “old” males, whereas in 2012, the proportion of “old” males increased to 51%. However, this comparison is based on pooled data. Unfortunately no data on yearly age structure from 2000–2004 is available and thus, the observed difference might be still within the year to year variation.

### Breeding success

**Figure 3:**
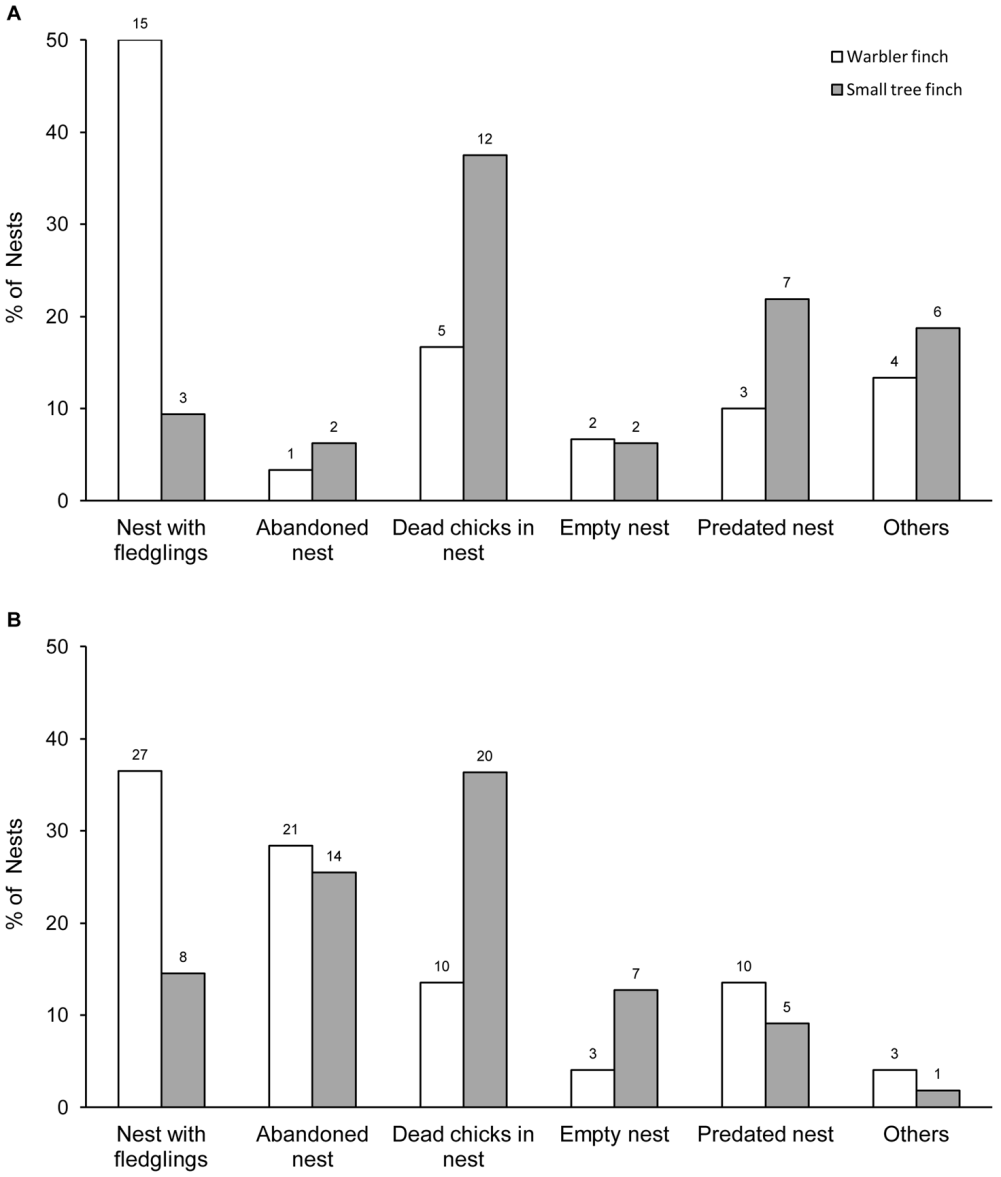
Breeding success and types of nesting failure. Proportional nesting outcome for the breeding season 2010 (A) and 2012 (B) of warbler finch nests and small tree finch nests of the *Scalesia* zone population on Santa Cruz, Galápagos. Numbers above bars indicate total numbers of cases.

In 2010, at least one chick fledged in 9% of the small tree finch nests, compared to 50% of the warbler finch nests (Figure 3a). The breeding success of small tree finches in 2012 was 16% compared to 37% of the warbler finches (Figure 3b). Breeding success was significantly higher for warbler finches than small tree finches in both years (Chi^2^-test, 2010: χ^2^_5, n = 62_ = 12.403, p = 0.001; 2012: χ^2^_5, n = 129_ = 7.682, p = 0.009; corrected α-level after Bonferroni: α = 0.025) and did not differ between the two years in both species (Chi^2^-test, warbler finch: χ^2^_1, n = 104_ = 1.619, p = 0.270; Fisher exact test, small tree finch: 3 out of 32 successful nests in 2010 and 8 out of 55 in 2012, p = 0.739).

**Figure 4:**
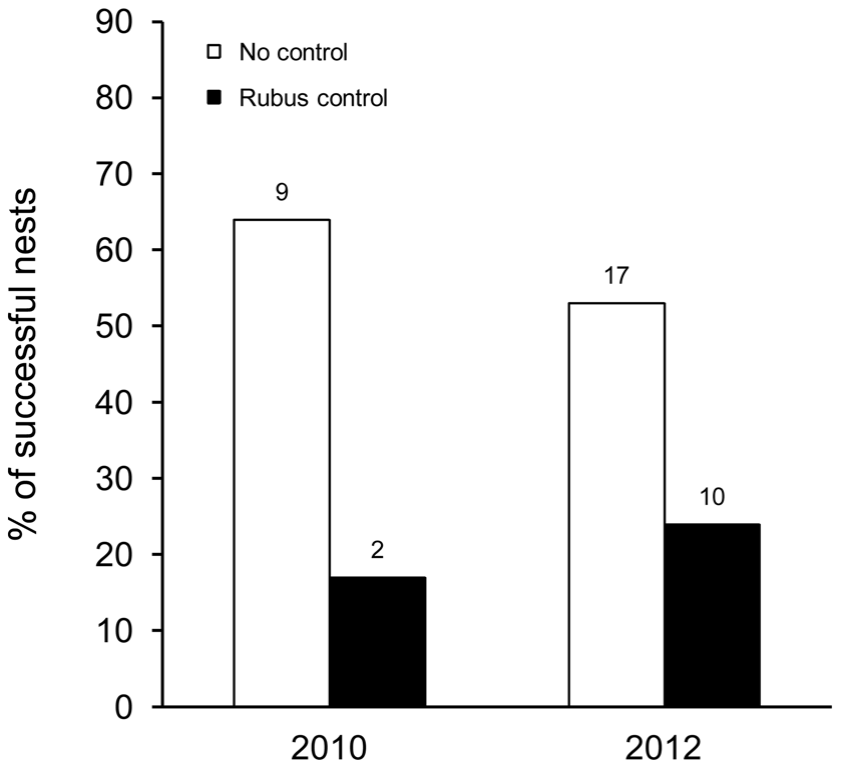
Effect of herbicide use on the breeding success of warbler finches. Percentage of successful warbler finch nests in areas with no control measures by the National Park (2010: n = 14, 2012: n = 32) and in areas where the National Park recently sprayed herbicides to control the invasive *Rubus niveus* (2010: n = 12, 2012: n = 42). Numbers above bars indicate total numbers of cases.

Breeding success was significantly influenced by *Rubus* control measures. In areas where the National Park had sprayed herbicides, breeding success of warbler finches was significantly lower than in areas that were not managed in both years (Chi^2^-test:, 2010: χ^2^_1, n = 26_ = 6.003, p = 0.021; 2012: χ^2^_1, n = 74_ = 6.735, p = 0.014, Figure 4). In small tree finches, 18% (n = 20) of the nests in the not controlled and none of the 15 nests of the controlled area were successful in 2010 and 11% (n = 21) of the nests in the not controlled and 17% (n = 42) of the nests in the controlled area were successful in 2012. Due to the overall low breeding success, a statistical analysis was not run for the small tree finch.

For both species, the majority of failed breeding attempts were assigned to the following three categories: “abandoned nest”, “predated nest” and “dead chicks in the nest” (Figure 3). However, reasons for failed breeding attempts differed between years (warbler finch: χ^2^_5, n = 104_ = 12.253, p = 0.029, small tree finch: χ^2^_5, n = 87_ = 15.253, p = 0.007). The post-hoc analysis revealed that this difference was due to a higher proportion of abandoned nests in 2012 (warbler finch: χ^2^_1, n = 104_ = 8.188, p = 0.007, tendency in small tree finch: χ^2^_1, n = 87_ = 4.813, p = 0.042, all other post-hoc comparisons n.s., corrected α-level after Bonferroni: α = 0.017).

**Figure 5:**
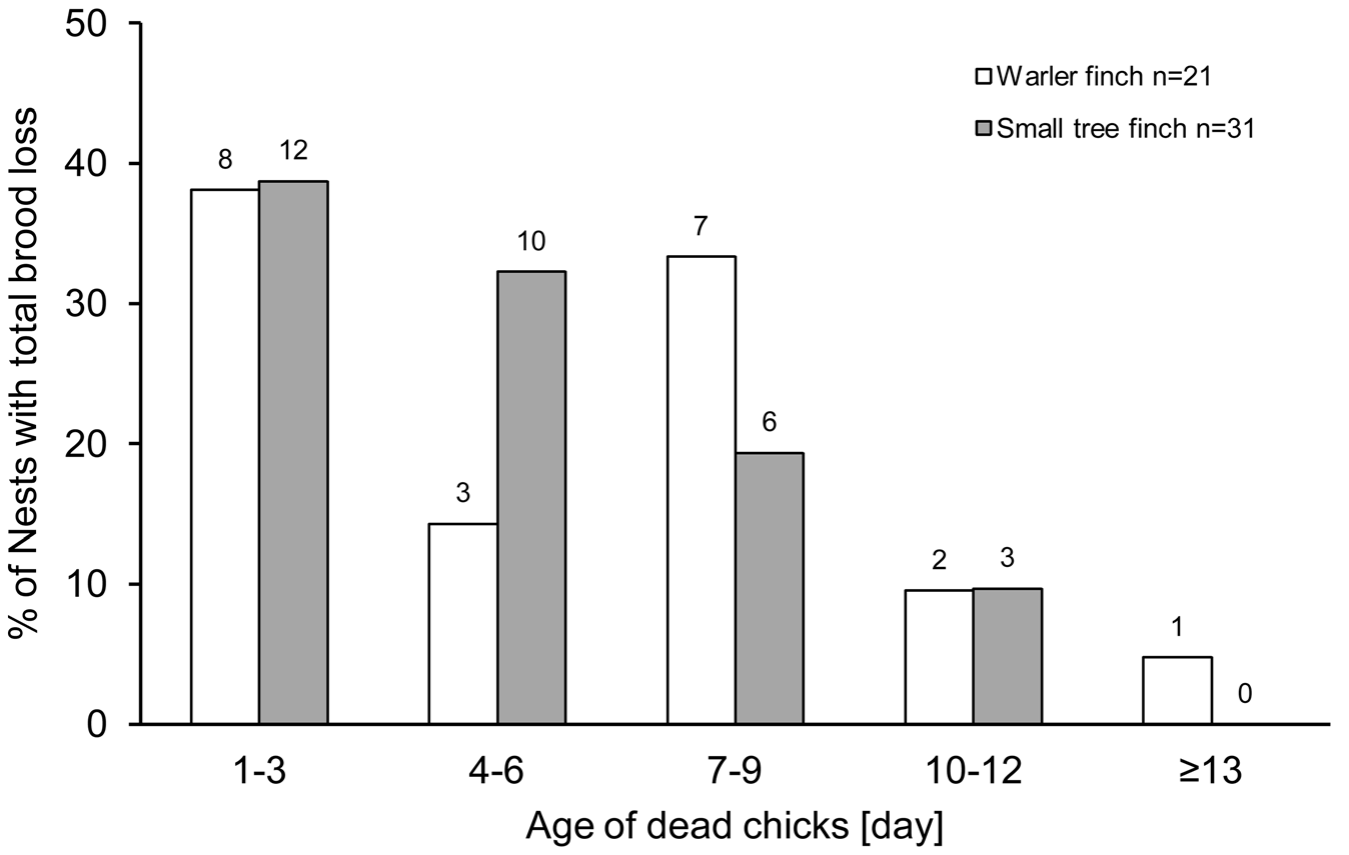
Age of chicks at brood loss. Percentage of nests with total brood loss depending on the chicks' age. Warbler finch (n = 21), small tree finch (n = 31). Total brood loss included the following types of nesting failures: dead chicks in the nest, empty nest and predated nests. Numbers above bars indicate total numbers of cases.

In 2012, most of the small tree finch nests that lost their total brood did so before the chicks reached 7 days (71%, Figure 5). In the warbler finch, two distinct age classes showed the highest percentage of nests with total brood loss (Figure 5): 38% failed when chicks were 1–3 days old and 33% failed when chicks were 7–9 days old. For both species, the main categories of breeding failure in the age class 1–3 days was “dead chicks in the nest” and “empty nest” (18 nests out of 20).

### Abandoned nests

In 2012, 21 warbler finch nests and 13 small tree finch nests were abandoned before the chicks hatched, thus still containing eggs (Figure 3). We found undeveloped eggs in 33% of abandoned warbler finch nests and in 19% of small tree finch nests, and chicks close to hatching, which did not manage to hatch, in 19% of abandoned warbler finch nests and in 15% of small tree finch nests. In both species, abandoned nests experienced a significantly higher percentage of heavy rain days during incubation than those in which chicks hatched (two tailed Student t-test: warbler finch t_67_ = 2.698, p = 0.009; small tree finch t_51_ = 2.129, p = 0.038).

### Predation and artificial nest experiment

Predation as a cause for nesting failure was observed in 22% of small tree finch nests in 2010 and 9% in 2012 as well as 10% of warbler finch nests in 2010 and 14% in 2012 (Figure 3). Potential predators observed in the study area were the short-eared owl (*Asio flammeus galapagoensis*, endemic), the smooth-billed ani (*Crotophaga ani*, introduced), and the black rat (*Rattus rattus*, introduced). The artificial nest experiment revealed similar results. Over the whole experimental period (9 days), eggs from 7 of the 30 artificial nests (23%) showed signs of predation at least once. There was no difference in predation rate between the *Rubus* controlled area (3 of 15 predated artificial nests) and the non-controlled area (4 of 15 predated artificial nests, Fisher exact test: p = 1.000). In total, 20 Plasticine eggs of the possible 180 eggs showed signs of predation. Of these eggs, 18 had clear rat tooth marks.

### Nesting failure and *P. downsi* infestation

A high percentage of nests contained dead chicks, though they showed no signs of predation (2010: warbler finch 17%, small tree finch 38%; 2012: warbler finch 14% and small tree finch 30% of the nests; Figure 3). Since all of these nests suffered high parasite loads and no other cause of death was found, we attributed these nesting failures to *P. downsi* parasitism. If we include the fledging nests that also contained dead chicks (partial brood loss), then 56% of small tree finch chicks and 37% of warbler finch chicks most likely died due to *P. downsi* parasitism in 2012 (data for 2010 not available).

We found 100% prevalence of *P. downsi* for nests which contained nestlings. However, the two finch species were affected differently. Small tree finches, the species with significantly lower breeding success, had significantly more *P. downsi* specimens per nest than the warbler finch (two-way ANOVA: species, F_1,125_ = 7.349, p = 0.008). There was no year effect (F_1,125_ = 1.912, p = 0.169) nor an interaction of year and species (F_1,125_ = 0.11, p = 0.916).

**Figure 6:**
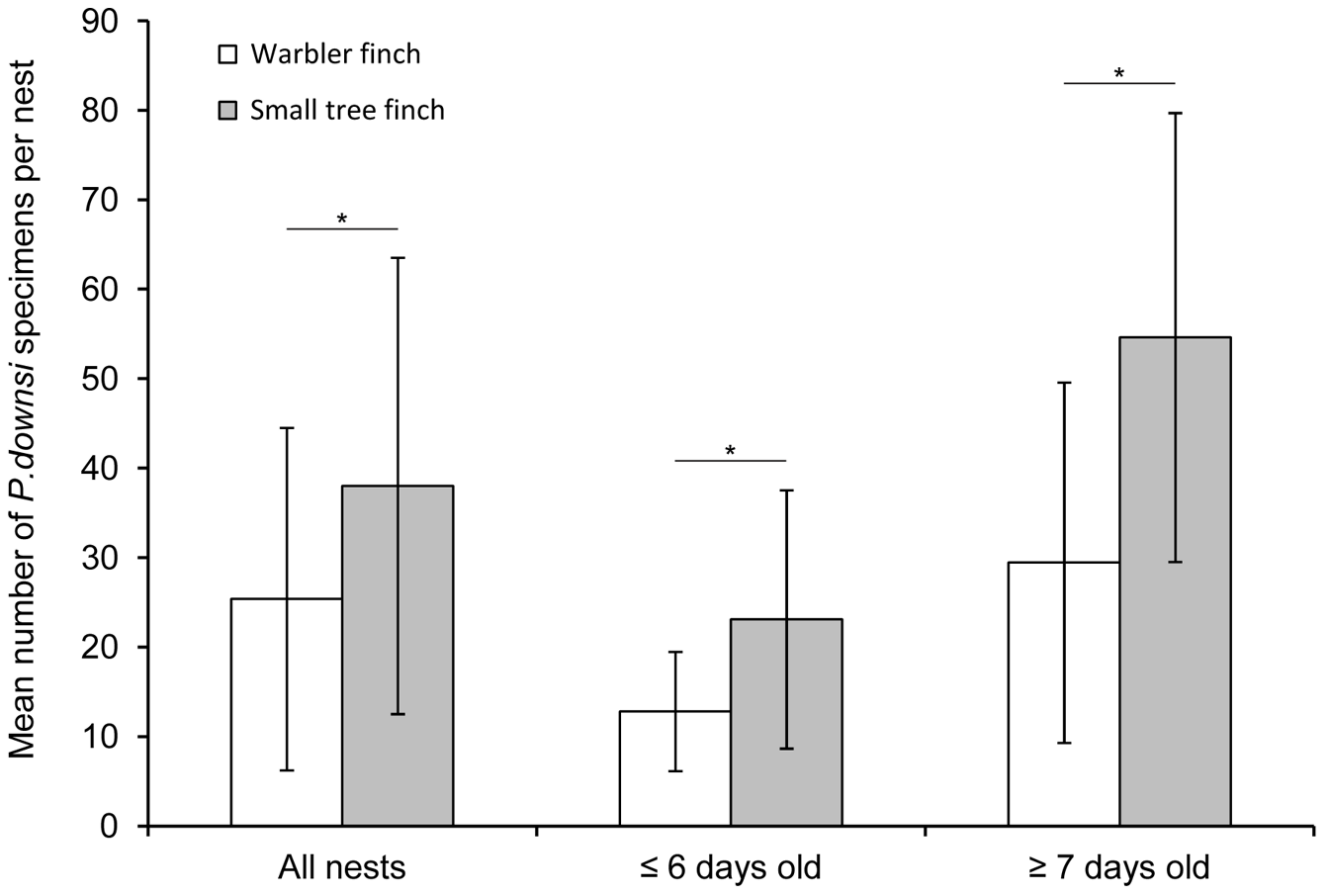
*Philornis downsi* intensity in warbler finch and small tree finch nests. Mean (± SD) number of *P. downsi* specimens (larvae, pupae and puparia) per nest of the breeding season 2012 of warbler finches and small tree finches for all nests with chicks (warbler finch n = 44, small tree finch n = 38), nests with chicks of six days and younger (warbler finch n = 11, small tree finch n = 20) and nests with chicks of seven days and older (warbler finch n = 33, small tree finch n = 18).

Because warbler finches had a higher breeding success than the small tree finch, mean nestling age was higher, too. As *P. downsi* intensity increases with nestling age [40], this could bias the data. Thus, we analysed nests with chicks of 6 days and younger separately to reduce the effect of nestling age. In 2012, significant differences in *P. downsi* intensity were already present in nests with chicks of 6 days and younger (Student t-test: *P. downsi*/nest, t_29_ = −2.222, p = 0.034; *P. downsi*/chick, t_29_ = −2.631, p = 0.014, Figure 6). They were also still present in nests with chicks of 7 days and older (two tailed Student t-test: *P. downsi*/nest, t_50_ = −3.934, p<0.001; *P. downsi*/chick, t_50_ = −4.295, p<0.001, Figure 6).

Because of the 100% prevalence of *P. downsi* larvae, we are not able to test whether a lower infection rate at early nestling stage results in a higher probability of a successful nest. However, parasitism may not be the only reason for breeding failure, and so we analysed whether other factors may also influence breeding success. In 2012, we found that the only factor to negatively influence breeding success significantly in both species was the percentage of heavy rain days during the nestling period (full and reduced model, Table 1). In warbler finches breeding success also increased later in the season (full and reduced model, Table 1).

**Table 1:** Variables that explained variation in breeding success of warbler finches and small tree finches in 2012 by GLMs with a binomial error structure.

Dependent variable: Breeding success		Warbler finch (n = 44)				Small tree finch (n = 33)			
	Predictors	Estimate	SE	z	P	Estimate	SE	z	P
*Full model*	Intercept	−4.79	4.07	−1.18	0.24	−2.94	10.54	−0.28	0.78
	**% Heavy rain days in nestling phase**	**−0.21**	**0.07**	**−3.15**	**0.002**	**−0.21**	**0.10**	**−2.19**	**0.03**
	% Heavy rain days in incubation phase	6.41	4.18	1.53	0.13	3.32	8.93	0.37	0.71
	Nest height	0.22	0.40	0.54	0.59	−0.12	0.32	−0.37	0.71
	Canopy cover	0.04	0.02	1.64	0.10	0.01	0.03	0.34	0.73
	**Date of incubation start**	**0.13**	**0.06**	**2.06**	**0.04**	0.14	0.19	0.75	0.46
*Reduced model*	Intercept	3.05	1.54	1.98	0.05	3.05	1.54	1.98	0.05
	**% Heavy rain days in nestling phase**	**−13.73**	**4.58**	**−2.99**	**0.003**	**−14.21**	**6.02**	**−2.36**	**0.02**
	**Date of incubation start**	**0.08**	**0.03**	**2.33**	**0.02**	-	-	-	-

Significant variables are marked in bold.

## Discussion

In summary, breeding success was decreased by *P. downsi* parasitism and heavy rain days in warbler finches and small tree finches while predation had only a minor influence. Additionally, the use of herbicides on the surrounding habitat had a significant negative effect on the reproductive performance of the warbler finch.

Although the direct cause of death could not be identified, it is very likely that parasitism by *P. downsi* played a major role in the death of 56% of small tree finch chicks and 37% of warbler finch chicks. Indirect evidence for the role of *P. downsi* comes from the comparison of breeding success in relation to parasite intensity between the small tree finch and the warbler finch: the nests of the small tree finch, the species which had significantly lower breeding success, had significantly higher *P. downsi* abundance. This difference was already present in the early nestling phase, in which mortality was particularly high. However, only experimentally manipulating parasite intensity can reveal the impact of *P. downsi* on mortality. In an experimental study on Darwin's ground finches where parasites were eliminated in the nests, mortality was reduced from 66% to only 14% [15] (but see [21]). In a similar study by Koop et al. [18], mortality decreased from 96% to 67% as parasite intensity was reduced.

The higher parasite loads and lower breeding success of small tree finch nests compared with warbler finch nests were contradictory to our predictions and to previous findings, which report higher *P. downsi* intensities in the warbler finch than in the small tree finch [17], [20]. In the small tree finch, the mean *P. downsi* intensity per chick (26.0±3.1, n = 18) for nests with nestlings of ≥6 days old was also higher than previously reported (mean *P. downsi*/chick: 20.5±2.3, n = 29 [20]). However, these comparisons are based on pooled data from 1998 to 2005 [20]. Since the Galápagos Islands are subject to massive environmental variation between years, and breeding density as well as parasite intensity might be affected by that, the average could smooth out annual variability. The pooled data from Dudaniec et al. [20] included one El Niño year (1998) and five years with low precipitation. Our data were collected in very humid years, which could explain the reversed infestation pattern. Findings on the relationship between parasite intensity, precipitation and host density are mixed (reviewed in [21]) and thus this relationship is not well understood so far.

The low breeding success of small tree finches may additionally be explained by limiting ecological conditions such as availability of suitable prey for chick rearing. In Darwin's ground finches, gonadal activity is triggered by rain [43] as insect abundance starts to increase about 10 days after the first rains on the Galápagos Islands [22]. It seems plausible that warbler finches and small tree finches similarly start to breed when the rain starts, but that they depend on prey [44] with different phenology. Preliminary data on breeding phenology suggests that small tree finches start breeding later and are more synchronic than the opportunistic warbler finches. Future studies focusing on breeding phenology and detailed foraging observations may highlight some key factors, such as special food sources, which have been overlooked so far.

In 2012, a high percentage of nests (small tree finch 25%, warbler finch 28%) were abandoned during incubation (before the chicks hatched). Studies on Darwin's finches on several other islands have previously reported much lower values (e.g. [12]: 1.8–6.7 %; [36]: 7%; [10]: up to 10%). However, O'Connor et al. [45] found 30.4% (data of 2004 and 2006 combined) of small ground finch nests abandoned in the lowlands, and in 2005, 25% abandoned nests in the highlands of Floreana Island. For 2005, it was suggested that extreme drought conditions lead to this high percentage of abandoned nests. Our data suggest that nest abandonment during the incubation stage may also be increased by heavy rain days. Intense rains may negatively affect nest temperature and humidity, thus leading to nest abandonment. This may be linked to higher energy costs for the incubating female, since clutches will cool more rapidly in a wet nest (reviewed in [46]). Thus, in both cases, extreme weather conditions are related to abandonment of the nest in the incubating stage. Heavy rains also affected breeding success of both species during chick rearing. During intense rain, parents may be less efficient in foraging or even stop searching for food as insects are more difficult to find. This temporary lower food abundance probably leads to lower feeding rates by the parents and thus poses stress on chicks, especially those that are very young and require regular feeding. This hypothesis should be investigated in future studies.

*Rubus* control with herbicides also negatively affected breeding success. Warbler finches had significantly lower breeding success in areas where herbicides had recently been applied compared with uncontrolled areas. The use of strong herbicides by the National Park to control the invasion of *R. niveus* leads to removal of more or less the entire understory (Cimadom personal observation). This may have lead to changes in abundance and composition of invertebrate species, which could negatively affect chick rearing, especially in insectivorous bird species. However, more data on chick food is needed, as it is very likely that more vegetarian bird species also depend on invertebrates for chick rearing. Additionally, the likely decrease in invertebrate abundance triggered by the use of herbicides might also negatively affect adult survival of insectivorous bird species, especially during the dry season, when arthropods are less common in general. The fact that four of the six declining species are insectivorous [27] supports this notion. It has already been shown that the use of herbicides especially glyphosate has a negative effect on abundance of several bird species [47], [48] and Betts et al. [49] found that especially leaf-gleaning bird species declined after herbicide treatment. Specifically open-cup nesting species had a significantly lower breeding success in herbicide-treated forest areas than in manually thinned areas [50]. However some management to restore the highly endangered *Scalesia* forest is indispensable: The high cover of the invasive *Rubus niveus* has lead to significantly lower native plant species richness and cover, as well as to changes in the *Scalesia* forest structure [51] which is likely to affect flora and fauna. The restoration of the *Scalesia* forest is an example of the conservation of a highly fragile ecosystem. Restoration measures might be positive for some species but negative for others. Thus, it is important to consider the whole ecosystem when assessing the costs and benefits of different management strategies.

The effect of predation on breeding success was lower than expected and lower than in previous studies from Santa Cruz (pooled data from 1998, 2000, 2001, 2002, 2004 and 2005, 50% of small tree finch and 37.1% of warbler finch nests were predated, [20], [36], [52]). These findings could either be attributed to differing definitions of depredated nests or to a decline in the main predators (rats and short eared owls). Possibly, these predators are affected directly or indirectly by intense plant control with herbicides. The results of the artificial nest experiment support the notion that predation in the *Scalesia* forest was low.

## Conclusions and future plans

We found three factors that negatively influenced the breeding success of both finch species: *P. downsi* parasitism, habitat change due to control of invasive plant species and adverse climatic conditions (days of heavy rain). We hypothesize that the combination and interaction of these factors, rather than one single factor, leads to breeding failures. For instance, the negative role played by *P. downsi* parasitism may be increased by occurrence of intense rainfall (natural stressor) and major habitat changes, leading to low breeding success. While parents might be able to compensate for energy losses due to parasitism under optimal conditions, they might be unable to overcome the negative effect of parasitism when additional stressors (e.g. heavy rain events, *Rubus* control) come into play. This hypothesis should be investigated in future studies on breeding success, for example by selectively manipulating parasite abundance through use of insecticides in areas with invasive plant species control and in uncontrolled areas. These data could then be combined with daily weather conditions.

It is still unclear whether the low breeding success measured in this study can explain the dramatic decline of the warbler finch population in the *Scalesia* forest on Santa Cruz (down 58% compared to 1997), as reliable population models are still missing. To date, there is no data on the age structure, juvenile survival, and dispersal behaviour of the warbler finch. Future studies should aim to collect this relevant information in order to be able to develop meaningful population models, which are helpful tools for evaluating possible conservation measures. In small tree finches, our data on a change in age structure and the extremely low breeding success in 2010 and 2012 go in parallel with a significant population decrease since 2005. Whether this decrease is due to natural fluctuation in population size or is the result of parasitism and habitat change needs to be assessed in future studies. Furthermore, more information regarding breeding ecology and foraging behaviour are needed to further explore the relationship between the three identified factors and their negative influence on breeding success.
